# ASSURED-optimized CRISPR protocol for knockout/SNP knockin in hiPSCs

**DOI:** 10.1016/j.xpro.2023.102406

**Published:** 2023-07-22

**Authors:** Katarzyna A. Ludwik, Narasimha Telugu, Sandra Schommer, Harald Stachelscheid, Sebastian Diecke

**Affiliations:** 1 Berlin Institute of Health at Charité – Universitätsmedizin Berlin, Core Unit Pluripotent Stem Cells & Organoids, 13353 Berlin, Germany; 2 Max-Delbrück-Center for Molecular Medicine in the Helmholtz Association (MDC), 13125 Berlin, Germany; 3 DZHK (German Centre for Cardiovascular Research) (partner site), Berlin, Germany

**Keywords:** Molecular Biology, CRISPR, Stem Cells

## Abstract

CRISPR-Cas9 technology coupled with human induced pluripotent stem cells allows precise disease modeling in pluripotent cells and subsequently derived specialized cell types. Here, we present an optimized CRISPR-Cas9 pipeline, ASSURED (affordable, successful, specific, user-friendly, rapid, efficient, and deliverable), to produce gene-modified single-cell-derived knockout or single-nucleotide-polymorphism-modified knockin hiPSCs clones. We describe steps for analyzing targeted genomic sequence and designing guide RNAs and homology repair template. We then detail the CRISPR-Cas9 delivery workflow, evaluation of editing efficiency, and automated cell isolation followed by clone screening.

## Highlights

• Complete workflow for generation of gene-edited iPSCs using CRISPR/Cas9
• Detailed guide to CRISPR targeting design
• Optimized protocol of CRISRP/Cas9 delivery and editing efficiency assessment
• Description of improved automated single-cell cloning and clone screening

## Abstract (editor highlights)

Publisher’s note: Undertaking any experimental protocol requires adherence to local institutional guidelines for laboratory safety and ethics.

## Graphical abstract

**Figure:**
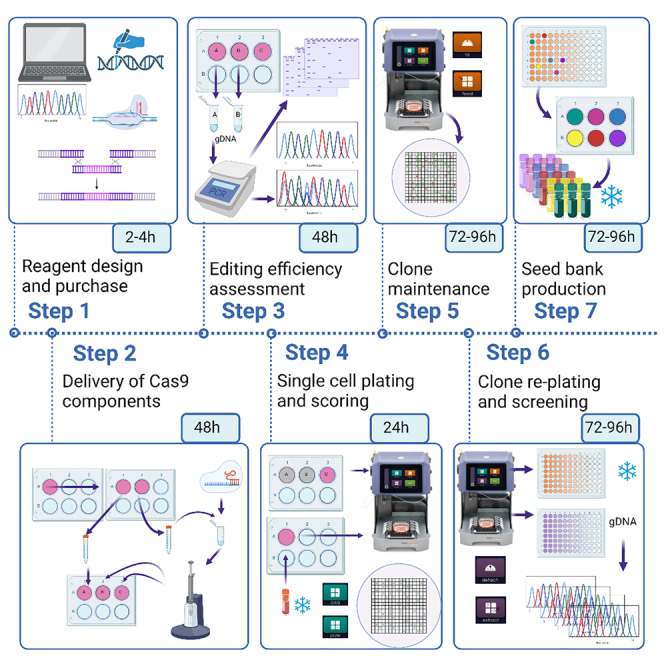

## Teaser

CRISPR-Cas9 technology coupled with human induced pluripotent stem cells allows precise disease modeling in pluripotent cells and subsequently derived specialized cell types. Here, we present a CRISPR-Cas9 pipeline to produce gene-modified single-cell-derived knockout or single-nucleotide polymorphism-modified knockin hiPSCs clones. We describe steps for analyzing targeted genomic sequence and designing guide RNAs and homology repair template. We then detail the CRISPR-Cas9 delivery workflow, evaluation of editing efficiency, and automated cell isolation followed by clone screening.

## Before you begin

The protocol below describes performing a gene knockout (KO) or single nucleotide editing (knockin, KI) in human iPSC or ESCs. We provide two examples: a knockout of the SorCS1 gene by targeting exon 4 and a knockin to introduce premature stop codon in exon 25 (H1098∗, c.3292_3294delinsTAG) of SorCS1. The modifications are carried out in hiPSC line BIHi005-A (https://hpscreg.eu/cell-line/BIHi005-A). The following protocol can be easily applied in other stem cell lines and for other genomic locations.

### Obtain gene sequence

**Timing: 15 min**

Here we describe briefly one of the several available methods for obtaining the gene sequence. It is beyond the scope of this protocol, but we highly recommend becoming familiar with the gene structure, genomic location and all possible isoforms of the gene of interest.

1. Register an account on Benchling to access your workspace (https://www.benchling.com/).
2. To import DNA sequences using either Gene name or ENSEMBL ID, click on ‘+’ sign on the left panel.
3. Select CRISPR from the drop-down menu.
4. Select CRISPR guides from the drop-down menu.
5. Input gene name or ENSEMBL ID.
6. For example: SorCS1 or ENSG00000108018.
7. Make sure that the selected genome is correct.
8. For example: Homo sapiens GRCh38.
9. Set the destination folder to save the DNA sequence by creating new project folder.
10. Click on ‘Import’ to obtain the annotated gene sequence.

***Note:*** Benchling will import the single transcript chosen for a gene which is the most conserved, highly expressed, has the long coding sequence and is represented in other key resources such as NCBI or Uniport. In case specific isoform information is required, or a knockout targeting all isoforms we recommend viewing gene information using GeneViewer on NCBI.

***Alternatives:***

11. Using ‘Gene’ search function in NCBI (https://www.ncbi.nlm.nih.gov/) input the gene of interest. For example, gene name: SorCS1.
12. Make sure that the selected gene is from species *Homo sapiens* and identify the gene_ID. For example, gene_ID for SorCS1: 114818.
13. Go to full gene report on NCBI (https://www.ncbi.nlm.nih.gov/gene/gene_ID).
14. Go to section with reference sequences (NCBI Reference Sequences (RefSeq)).
15. Identify the genomic sequence. For example, annotation for SORCS1, gene_ID 114818, GenBank:NG_029120.

***Note:*** the annotation of the full gene sequence will be a range of a chromosome sequence and should start with letters ‘NG’.

16. Download GeneBank or FASTA file.

***Note:*** choose the file format depending on your preferred sequence browser, i.e., SnapGene etc.

17. Using sequence viewer program (i.e., SnapGene or Benchling) identify the exons, introns, CDS, and protein translation.

***Note:*** other useful tools for viewing gene structure include: Ensemble (https://www.ensembl.org/index.html) and UCSC Gene Browser (https://genome.ucsc.edu/).

### hiPSC culture

**Timing: 1 week**

Ensure that hiPSC cells used for gene editing are of excellent quality. Use the earliest available passage and make sure that the standard quality control has been completed. Each of our cell banks is subject to g-banding karyotyping and SNP analysis to test genetic stability, mycoplasma and sterility testing, morphology assessment and STR analysis to confirm cell line identity.^1^ Additionally, we recommend testing for expression of markers for the undifferentiated state of hPSC (i.e., Oct3/4, Nanog, SSEA1, Tra-1-60) using immunofluorescence or FACS.

For our maintenance culture we routinely use Geltrex coating and Essential 8 media. However, certain cell lines might have specific media and matrix recommendations, i.e., mTeSR or StemFlex media, and vitronectin or Matrigel coating. During the process of gene editing, we switch from standard maintenance media to StemFlex or StemMACS (see key resources table), as we observe improved recovery after nucleofection and clone survival in these formulations.

Genetic engineering places the cells under selection pressure and requires several rounds of cell doublings until the edited clone is obtained. Therefore, to avoid accumulation of genomic instabilities, we recommend using cells 2–3 passages after thawing as close to the quality tested cell bank as possible. We also recommend maintaining hiPSCs and performing gene editing experiments in hypoxic conditions (37°C/5% CO_2_/5% O_2_) as culturing hiPSCs under hypoxic conditions has several advantages, including enhanced pluripotency, increased proliferation, reduced oxidative stress, improved reprogramming efficiency, better differentiation potential and low frequency of genetic instabilities.^2^^,^^3^ These benefits can result in improved hiPSC quality and functionality, which are crucial for downstream applications in regenerative medicine and disease modeling. General protocols describing plate coating, cell maintenance, and enzymatic and non-enzymatic dissociation have been described by Vallone et al.^4^

### Geltrex dilution and aliquots

**Timing: 1 h**

18. Thaw Geltrex stock solution on wet ice at 4°C.

**CRITICAL:** The following steps must be performed on ice! All serological pipettes and tubes to be used should be pre-chill at −20°C for at least 20 min.

19. Transfer 45 mL of KO DMEM/F-12 to a 50 mL conical tube.
20. Add 5 mL of thawed Geltrex stock solution to the aliquoted KO DMEM/F-12.
21. Mix gently without introducing bubbles.
22. Quickly aliquot 1 mL into 1.5 mL Eppendorf tubes.
23. Place on ice immediately.
24. Transfer the aliquots into −20°C as soon as aliquoting is finished.

These aliquots must be further diluted immediately prior to application 1:12 in KO DMEM/F-12 to generate Geltrex Coating Solution.

### gDNA isolation

**Timing: 1 h**

Genomic DNA isolated from the parental cell line will be necessary for primer optimization, confirmation of genotype and identification of potential nucleotide polymorphisms in the sgRNA and HDR regions. Therefore, we recommend preparing this gDNA in advance from all the cell lines that will be subject to the genetic modification. It is possible to use gDNA isolated by crude methods (i.e., Phire Tissue direct kit or similar), but isolation on silica based columns or beads is preferable. We routinely use Qiagen and Promega DNA isolation kits.

## Key resources table

**Table:** 

REAGENT or RESOURCE	SOURCE	IDENTIFIER	
**Chemicals, peptides, and recombinant proteins**			

StemFlex media	Life Technologies	A3349401	
StemMACS™ PSC-Brew XF, human	Miltenyi Biotech	130-127-865	
StemMACS PSC-Support XF, human	Miltenyi Biotech	130-127-287	
CloneR2	Stem Cell Technologies	100-0691	
Geltrex	Thermo Fisher Scientific	A1413302	
KO DMEM/F-12	Thermo Fisher Scientific	12660-012	
PBS (-Ca/-Mg)	Thermo Fisher Scientific	14190144	
TrypLE	Life Technologies	12563011	
Cell culture grade water	Corning	25-055-CM	
0.5 M EDTA, pH 8.0	Thermo Fisher Scientific	15575020	
E8 medium	Thermo Fisher Scientific	A1517001	
iMatrix-511 Recombinant Laminin E8 Fragments	AMSBIO	AMS.892 011	
Bambanker	Nippon Genetics	BB01	
Pierce™ 20× TBS Buffer	Pierce	28358	
Roti ® - GelStain	Carl Roth	3865.2	
Alt-R® S.p. HiFi Cas9 Nuclease V3, 500 μg	Integrated DNA Technologies	1081061	
Alt-R™ HDR Enhancer V2, 150 μL	Integrated DNA Technologies	10007921	

**Critical commercial assays**			

Neon™ Transfection System 10 μL Kit	Thermo Fisher Scientific	MPK1096	
Phire Tissue Direct PCR Master Mix	Thermo Fisher Scientific	F170S	
DNeasy Blood & Tissue Kit (250)	Qiagen	69506	
GeneJET PCR Purification Kit	Thermo Fisher Scientific	K0702	
GeneJET Gel Extraction and DNA Cleanup Micro Kit	Thermo Fisher Scientific	K0832	

**Experimental models: Cell lines**			

BIHi005-A, iPSCs	Berlin Institute of Health	https://hpscreg.eu/cell-line/BIHi005-A	

**Oligonucleotides**			

Exon4_Pair1_Fwd CATCCCTCGTTCTTCCAGCTT	This paper	N/A	
Exon4_Pair1_Rev TATTTTCCCCGGAGAACCATGC	This paper	N/A	
Exon4_Pair2_Fwd TTTCATCCCTCGTTCTTCCAGC	This paper	N/A	
Exon4_Pair2_Rev GCTCACCTTTTGGTCTTGACTG	This paper	N/A	
Exon25_Pair1_Fwd GATGCATCCCATGTCAGCCA	This paper	N/A	
Exon25_Pair1_Rev TTCTCATTCTGCATCTGGGCA	This paper	N/A	
Exon25_Pair2_Fwd TGCTTTTCATTGCTCTGCTGC	This paper	N/A	
Exon25_Pair2_Rev TGCACATCACTGTAACACATGC	This paper	N/A	
Alt-R® CRISPR-Cas9 gRNA, 2 nmol SorCS1-Exn4-gRNA1 GATAATGTTACTCACAGACC	This paper; Integrated DNA technologies (IDT)	IDT predesigned and custom gRNA	
Alt-R® CRISPR-Cas9 gRNA, 2 nmol SorCS1-Exn4-gRNA2 ATAAACTGCTCTCAATCTCC	This paper; Integrated DNA technologies (IDT)	IDT predesigned and custom gRNA	
SorCS1-Exn25-gRNAs and ssODN, see Table S2	N/A	N/A	

**Software and algorithms**			

Gene retrieval, ssODN design, gRNA design	Benchling	https://www.benchling.com/	
Gene retrieval	ENSEMBLE	https://www.ensembl.org/index.html	
Gene retrieval	NCBI	https://www.ncbi.nlm.nih.gov/gene	
gRNA Design	Synthego	https://design.synthego.com/#/	
Primer design	NCBI primer design tool	https://www.ncbi.nlm.nih.gov/tools/primer-blast/	
ICE analysis	Synthego ICE analysis tool	https://ice.synthego.com/#/	

## Materials and equipment

### Alt-R® S.p. HiFi Cas9 nuclease V3 aliquots

Cas9 Nuclease from IDT is provided as 10 μg/μL solution. We recommend making 2 μL aliquots to avoid repeated thaw/freeze cycles.

### StemFlex/CloneR2

During the cell cloning stage of the protocol, we recommend using StemFlex media supplemented with CloneR2 (StemFlex/CloneR2) to increase cell survival. CloneR2 promotes clonal survival and growth of hiPSCs during single-cell passaging. CloneR2 improves the efficiency of colony formation from single cells while maintaining the pluripotent state.

CloneR2 is diluted in media 1:10, for example, for 18 mL of StemFlex add 2 mL of CloneR2. Media supplemented with CloneR2 should be used up as soon as possible. Therefore, for economic reasons, we recommend preparing StemFlex/CloneR2 always fresh and in quantity specifically required by the application that is being performed on that day.

Similar results were obtained with StemMACS PSC-Brew XF human supplemented with StemMACS PSC-support XF human. Essential 8 media can also be used during gene editing protocol; however, we experienced reduced cell survival and clone recovery.

## Step-by-step method details

### Design of guide RNAs

**Timing: 30 min**

CRISPR can be used to generate gene knockouts (KO), single nucleotide (SN) modification, and insertions/replacement of larger DNA fragments (knockin, KI).^5^ A major difference in design approach for these three applications is the selection criteria for gRNAs.

For SN editing and KIs, the gRNAs must be as close to the targeted site as possible. Therefore, the choice of potential gRNAs is frequently limited. On the other hand, gRNA selection for KOs is more flexible. In most cases, KOs can be generated through introduction of a single double-strand break (DSB) with a single gRNA or by using two gRNA near each other. Following DSB, non-homologous end joining (NHEJ) leads to random indel formation, with potential frame shift and, as a result, a premature stop codon occurrence or nonsense protein sequence. For a successful knockout, gRNA target sites must be in exons critical for protein function. Specifically, gRNA-target sequences near the N-terminus should be excluded to avoid usage of an alternative start-site downstream of the annotated start codon. Similarly, gRNA-target sequences near C-terminus ought to be avoided, to maximize the chance of frame shifts and generation of a non-functional protein.

We suggest using Benchling and/or CRISPOR online tools for gRNA design for all applications. However, when a non-targeted KOs is required, we recommend the more straightforward Synthego tool. The following steps describe design of gRNAs using online tools.

***Note:*** As online tools change constantly and rapidly, we provide just an overview of the steps. Most online tools are self-explanatory and interactive. We encourage the users to explore the manuals and FAQ sections to find additional information. Below we describe design of gRNAs to create indels, and SNPs insertion in SORCS1 gene in Benchling.

1. In the gene viewer in Benchling, select CRISPR icon on the right side (crosshair icon).
2. Select: Design CRISPR guides.
3. Use default settings:
  a. Design type: single guide.
  b. Guide length: 20bp.
  c. Genome: GRCh38 (hg38, Homo sapiens).
  d. PAM; NGG.
4. Click on Finish.
5. Select target region.

**Figure 1:**
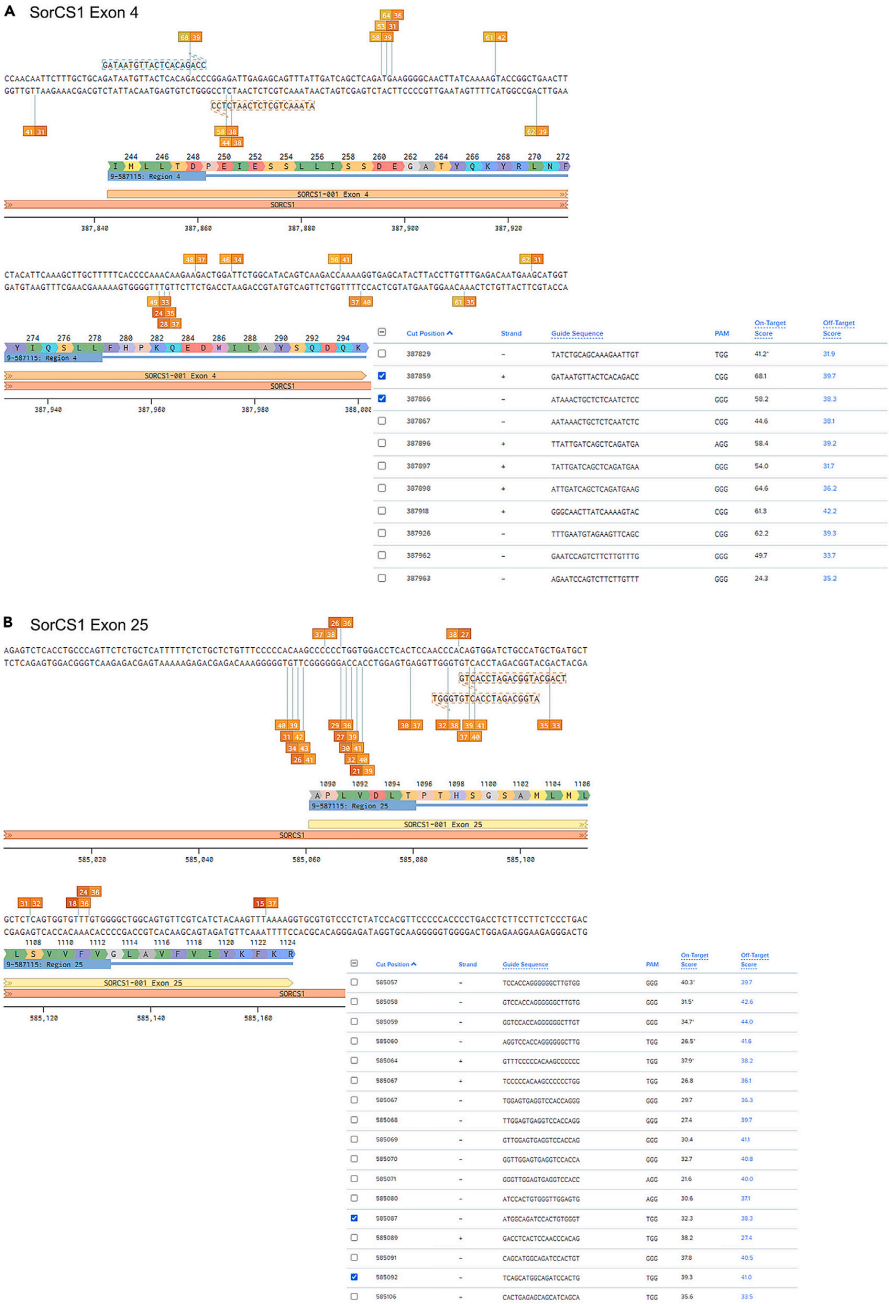
How to choose gRNAs (A) Structure of SorCS1 exon 4 with indicated protein translation, all guides available in the region, and a pair of guides selected for the KO. (B) Structure of SorCS1 exon 25 with indicated protein translation, all guides available in the region, and a pair of guides selected for the KI. Created with Benchling.com.

For example: exon 4 of SORCS1 for KO (Figure 1A) or exon 25 for SNP (Figure 1B).

***Note:*** make sure to select ∼30 bases before and after coding sequence to capture all the gRNA available in the region

6. Click on ‘+’ to create the guide sequences.
7. Benchling will generate a list of gRNAs available in the selected region with On-Target and Off-Target score values (Figures 1A and 1B).

**Optional step:** If working with CRISPOR, follow the below steps.

8. Input the sequence in the designated window.
9. Select genomic assembly: GRCh38.

***Note:*** Genome assemblies are updated continuously, thus, make sure to use the latest version.

10. Select PAM format: NGG.
11. Select gRNA length: 20bp.
12. Prompt the job submission.
13. The gRNA design tool will provide the list of gRNAs.

**Alternative:** If gRNAs are for KOs and the specific target region does not need to be considered, we recommend using the Synthego CRISPR Design Tool. This tool maximizes knockout probability by targeting an early part of the gene in an exon common to all transcript variants. All gRNAs are ranked according to their descending on-target activity and ascending off-target potential. In principle, one gRNA is sufficient to efficiently introduce an indel. However, *in silico* tools are still limited in predicting the cutting efficiency of gRNAs.^6^ Therefore, we recommend choosing 3–4 top scoring gRNAs and testing them *in vitro*.

### Selection of gRNAs

**Timing: 30 min**

We recommend selecting multiple gRNAs in the proximity of the desired modification. For KOs, up to 4 gRNAs can be used at the same time to increase the chance of indel formation. For KIs, use of 2 gRNAs simultaneously can increase probability of HDR.^7^ However, increasing number of gRNAs also increases potential off-target effects and in case of KIs can require introduction of additional silent mutations in the repair template. Therefore, the number of gRNAs used per modification should be carefully considered and will depend on the specific gene and targeted region. In the section delivery of CRISPR components to iPSCs, we describe delivery of 1 or 2 gRNAs.

14. For KI, assess all the gRNAs in the proximity to the position/SNP you aim to modify.
15. Select 2 gRNAs that (Figures 1A and 1B):
  a. have high on-target score;
  b. have minimal off-targets;
  c. the CRISPR site (guide sequence and PAM) overlaps the desired nucleotide change preferably in the seed region (Figure 2A) of the guide RNA (1–10 bases upstream of the PAM site;) or in one of the two G residues of the ‘NGG’ PAM site.

**Figure 2:**
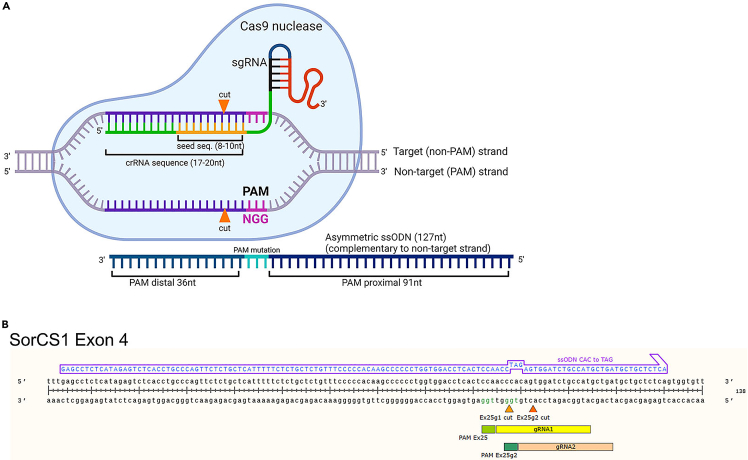
CRISPR/Cas9 RNP components (A) Schematic representing elements and orientation of gRNA and ssODN (HDR template) to target DNA locus. Created with BioRender.com. (B) Example alignment of two gRNAs and ssODN for introduction of premature stop codon in exon 25 of SorCS1 gene (H1098∗, c.3292_3294delinsTAG). Created with SnapGene.
  d. the cut site is as close to the desired SN as possible.
16. To increase a chance of obtaining heterozygous KI select the gRNAs 10bp upstream or downstream of SNP position.^8^

***Optional:*** step:

17. For KOs, select gRNA as in steps 15a-b and:
  a. A or T at the 4th position upstream of the PAM;
    ***Note:*** this will increase the probability of A or T duplication.^9^
  b. G at 1st position upstream of the PAM sequence;
    ***Note:*** gRNAs with G at 1st position have higher cutting efficiencies as compared to gRNAs with T at this position.^10^
  c. high out-of-frame and indel scores, if available in the online tool.

***Note:*** It is not always possible to fulfil all the above criteria for gRNAs.

**CRITICAL:** If the SNP is located outside of the seed region or corresponds to the ‘N’ of the ‘NGG’ PAM site, then the modified allele after HDR will be subject to re-cleavage by Cas9 RNP. Therefore, silent mutations should be introduced to either seed sequence or PAM sequence in the single-stranded oligodeoxynucleotide (ssODN) template to prevent recutting. When these additional mutations are not introduced, the number of screened clones needed to obtain the wanted modification can increase substantially. For details see section: design of ssODN for KIs.

### Design of primers

**Timing: 30 min**

As each hiPSC line is derived from an individual donor, there is a high probability of SN variations (SNV) occurrence in any given DNA region (Figure S2). Therefore, we recommend performing Sanger sequencing of the PCR-amplified targeted region before proceeding with gRNAs and ssODNs ordering. The primers used for this initial query can also be used for the future verification of the gene editing success and for clone screening.

18. Select approximately 250bp up- and downstream sequences from the target site.

***Note:*** Large deletions that expand past the fragment covered by the primers are possible as described by Simkin et al.^11^ Therefore, it might be required to use a primer pair spanning a larger region around target site. However, generation of hemizygotes rather than homozygotes is less likely to occur when RNPs are used, rather than plasmids.

19. Use Primer Blast design tool (https://www.ncbi.nlm.nih.gov/tools/primer-blast/) to design primers that will amplify 400–450bp of DNA with cut site position as close to the middle.
20. Select and order two primer pairs (Table 1, Figures S1A–S1B).

**Table 1:** PCR Primers for Exon 4 and Exon 25 regions of SorCS1 gene

Primer name	Sequence from 5′ to 3′	PCR product size (bp)
Exon4_Pair1_Fwd	CATCCCTCGTTCTTCCAGCTT	445
Exon4_Pair1_Rev	TATTTTCCCCGGAGAACCATGC	
Exon4_Pair2_Fwd	TTTCATCCCTCGTTCTTCCAGC	400
Exon4_Pair2_Rev	GCTCACCTTTTGGTCTTGACTG	
Exon25_Pair1_Fwd	GATGCATCCCATGTCAGCCA	428
Exon25_Pair1_Rev	TTCTCATTCTGCATCTGGGCA	
Exon25_Pair2_Fwd	TGCTTTTCATTGCTCTGCTGC	413
Exon25_Pair2_Rev	TGCACATCACTGTAACACATGC

***Note:*** To accurately estimate gene editing efficiencies and further select edited clones the quality of the PCR product and Sanger sequencing must be high. Therefore, we recommend choosing at least two primer pairs.

### Design of ssODN for KIs

**Timing: 30 min**

To facilitate specific SNP insertions, double strand breaks (DSB) generated by Cas9 can be repaired by an endogenous cellular homology-directed repair (HDR) pathway in the presence of donor template. We recommend using single-stranded oligodeoxynucleotides (ssODNs) as HDR repair templates. They can be synthetically produced, easily ordered, and handled. The following steps describe the design of HDR template recommended by *Richardson* et al.^12^.

21. Design HDR template 127nt in length.

***Note:*** increasing template length can improve HDR efficiency, this decision should be made based on the gene structure and desired modification, i.e., high repeat regions might need a bit longer homology arms.

22. We recommend HDR template to be asymmetric: 36nt distal homology arm (HA) and 91nt proximal HA (Figures 2A and 2B).^12^^,^^13^^,^^14^
23. Use target strand (non-PAM strand) as HDR template i.e., reverse complementary to the gRNA sequence (Figures 2A and 2B).
24. If possible, mutate PAM sequence in ssODN.

**CRITICAL:** It is critical to prevent Cas9 nuclease from recutting the modification site after the HDR. The strategy will depend on the choice of gRNA and the SNP.^15^^,^^16^^,^^17^ For example, if the SNP to be introduced already lies in the “GG” of the PAM, then additional mutations might not be necessary, since the recutting post HDR will be limited. If not, a silent mutation of codons covering the PAM sequence (one of the GG) should be introduced if possible; alternatively, the last five nucleotides of seed sequence can be mutated to prevent gRNA from binding.^18^^,^^19^^,^^20^^,^^21^^,^^22^

Below, we describe the design of ssODN using Benchling.

25. In the gene viewer in Benchling, select CRISPR icon on the right side (crosshair icon).
26. Select: Open HR template design and follow prompts.
27. The program will create the copy of the sequence.
28. Following the prompts in the right-side panel, insert the SNP sequence to be knocked in.
29. Adjust the HA as described in step 22.
30. Click **Next**.
31. The program will suggest mutations to the DNA template to prevent sgRNA from binding and recutting.

***Note:*** It is not always possible or desirable to introduce additional mutations. When designing HDR template without help of professional software (i.e., Benchling), we recommend to consult codon usage tables when introducing silent mutations. For example: https://www.genscript.com/tools/codon-frequency-table

32. Select the non-PAM strand as donor template as described in step 23.

***Note:*** Here we described template design for introduction of 1–5 base pair changes. However, it is also possible to introduce small tags using ssODN as templates. We have successfully integrated double HA (∼60bp) and triple Flag tag (∼70bp) at endogenous loci using ssODN templates. In these cases, the total template length was extended to 140 and 170 bp respectively.

### PCR optimization

**Timing: 8 h**

Due to unpredictability of PCR efficiency at different genomic loci we recommend testing both primer pairs designed in Steps 18–20 with at least three different polymerases i.e., PHIRE, AmpliTaq, and/or Kappa2G, and/or GXI polymerase, and/or Q5) at three annealing temperatures. Use gDNA from before you begin section: gDNA isolation.

33. Prepare 65 μL (3 × 20 μL rxns + 5 μL dead volume) of master mix for each polymerase and primer pair according to the polymerase specifications (Table 2, Table 3, Table 4), total 6 master mixes: 2 primer pairs × 3 polymerases.

**Table 2:** Volumes of reaction components for PHIRE polymerase

Reagent	Final conc.	Amount (1rxn) [μL]	Amount (3.5rxn) [μL]
Primer 1 (10 μM)	0.5 μM	1	3.5
Primer 2 (10 μM)	0.5 μM	1	3.5
2× PHIR taq buffer	1×	10	35
PhirTaqpolymerase∗	1×	0.5	1.75
gDNA	100 ng/μL	1	3.5
ddH_2_O	N/A	6.5	22.75
Total	**N/A**	**20**	**70**

**Table 3:** Volumes of reaction components for AmpliTaq polymerase

Reagent	Final conc.	Amount (1rxn) [μL]	Amount (3.5rxn) [μL]
Primer 1 (10 μM)	0.5 μM	1	3.5
Primer 2 (10 μM)	0.5 μM	1	3.5
2× Amplitaq PCR Mix	1×	10	35
GC enhancer	1×	1	3.5
gDNA	50–100 ng/μL	1	3.5
ddH_2_O	N/A	7	24.5
Total	**N/A**	**20**	**70**

**Table 4:** Volumes of reaction components for Q5 polymerase

Reagent	Final conc.	Amount (1rxn) [μL]	Amount (3.5rxn) [μL]
Primer 1 (10 μM)	0.5 μM	1	3.5
Primer 2 (10 μM)	0.5 μM	1	3.5
5× Q5 PCR buffer	1×	4	14
dNTP mix (2mM each)	0.2 mM	2	7
polymerase∗	1×	0.5	1.75
gDNA	50–100 ng/μL	1	3.5
ddH_2_O	N/A	10.5	36,75
Total	**N/A**	**20**	**70**
34. Aliquot 20 μL of each polymerase/primer pair master mix into 3 PCR tubes, total 3 × 6 master mixes: 18 tubes.
35. Use one tube from each polymerase/primer master mix for 3 different annealing temperatures (Table 5). Set up the thermocycler for respective conditions as in example (Table 6)

**Table 5:** Annealing temperature to be tested for different polymerases during primer optimization

Tube	Polymerase	Primer pair	Annealing temp. [C]
1	PHIRE	Pair 1	58
2			60
3			62
4		Pair 2	58
5			60
6			62
7	AmpliTaq	Pair 1	58
8			60
9			62
10		Pair 2	58
11			60
12			62
13	Q5	Pair 1	58
14			60
15			62
16		Pair 2	58
17			60
18			62

**Table 6:** Example thermocycler settings for DreamTaq polymerase reactions

Steps	Temperature	Time	Cycles
Initial Denaturation	95°C	1–3 min	1
Denaturation	95°C	30 s	40
Annealing	58/60/62°C	30 s	
Extension	72°C	1 min	
Final extension	72°C	5–15 min	1
Hold	4°C	Forever
36. Analyze PCR reactions by agarose (1%–2%) gel electrophoresis.
37. Choose the condition with the clearest band and no off-target bands. For example, for each primer pair in SorCS1 exons 4 and 25 the clearest bands were obtained with PHIRE polymerase with 62°C annealing temperature (Figures S1C–S1D).
38. Repeat the PCR with the chosen condition.
39. PCR purify or gel extract the PCR product.

***Note:*** Choose any of the standard PCR or gel extraction methods that provide sufficient purity for Sanger sequencing.

40. Perform Sanger sequencing on the PCR product with forward primer, the same one used for PCR.
41. Analyze the sequencing by comparing the obtained sequence with the chosen genome reference.

***Note:*** As hiPSC lines are derived from various individual donors, it is not unlikely to find single nucleotide polymorphisms which could interfere with gRNA binding sites (Figure S2).

### Synthesis of CRISPR components

**Timing: 1–2 weeks**

Once the target sequence is analyzed and no SNP interfering with the gRNA and HDR template are detected, CRISPR components can be purchased. They are readily available from many vendors, we routinely obtain HiFi Cas9, gRNAs, and ssODNs from IDT (https://eu.idtdna.com/pages/products).

**CRITICAL:** The following steps describe the procedure using the components from IDT, when using a different source of gRNAs and ssODN templates follow the manufacturer instructions for reconstitution and storage.

42. Order 2–10 nmol of 2 gRNAs selected in step 15 and step 17.

***Note:*** The amount of sgRNA ordered depends on application and number of editings to be performed. 2 nmol is usually sufficient for up to 10 editings.

43. Order 2–10 nmol of AltR-HDR template (Lab ready 100 μM in IDTE, pH 8.0) selected in step 32.

***Note:*** The amount of HDR template ordered depends on application and number of editings to be performed. 2 nmol is usually sufficient for up to 50 editings.

44. Upon receiving lyophilized gRNAs, centrifuge the gRNA tubes.
45. For 2 nmol of gRNA add 20 μL of IDTE buffer to obtain 100 μM stock solution.
46. Store all CRISPR components at −20°C.

### Plate cells for nucleofection

**Timing: 0.5–1 h**

**Figure 3:**
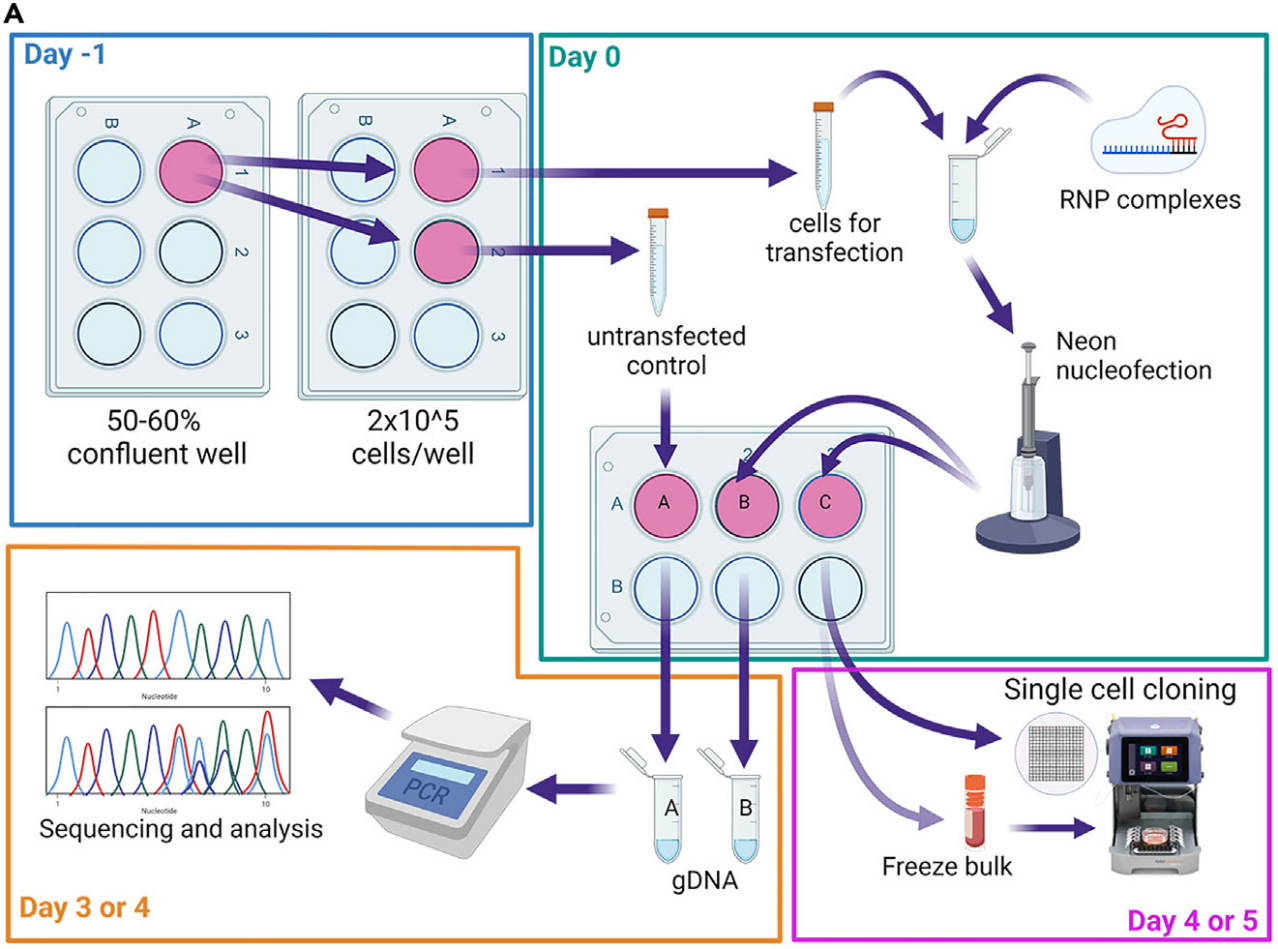
Workflow for cell preparation, nucleofection, screening, and cloning (A) Workflow representing cell preparation, nucleofection, bulk screening and single cell cloning (Steps 47–93). Created with BioRender.com.

The day before delivery of CRISPR components (Day -1), the cells from the section before you begin*: hiPSC culture* must be re-plated (Figure 3). This step helps to synchronize the cell cycle and promote most of the cell to be in S-phase, when HDR pathways are most active.^23^ The number of wells needed to begin the experiment is dependent on the number of nucleofections performed. Enough cells to plate 2 wells of a 6WP with 2 × 10^5^ cells each (one for one nucleofection and one for untransfected control) is needed (Figure 3). Usually one ∼50% confluent well of hiPSCs is enough to collect ∼1 × 10^6^ cells. However, we recommend evaluating cell number per well before performing the experiment.

47. Add 1 mL of Geltrex Coating Solution (see materials and equipment section) to each of 3 wells of a 6WP (receiving plate).
48. Incubate for 1 h at 37°C/5% CO_2_ incubator.
49. Prepare 10 mL of StemFlex/CloneR2 (see materials and equipment section), keep at RT.
50. Passage the cells from one ∼50% confluent well of a 6WP (cultured as recommended in before you begin: hiPSC culture section; Figure 3):
  a. Wash the cells once with room temperature (RT) PBS (-Ca, -Mg).
  b. Add 1 mL of TryplE to a well of 6 well plate.
  c. Incubate for 6–7 min at RT.
  d. Add 2 mL of StemFlex/CloneR2 per well.
  e. Triturate the cells for 3–5 times with P1000.
  f. Collect the cells in 15 mL falcon tube.
  g. Centrifuge the cells at 300 g for 5 min.
  h. Aspirate the media.
  i. Resuspend the cells in 1 mL of StemFlex/CloneR2 media by gentle pipetting with P1000.
  j. Perform cell count.
51. Aspirated Geltrex Coating Solution from the wells.
52. Plate 2 × 10^5^ cells per well in 2 wells of a 6WP in total volume of 1.5 mL of StemFlex/CloneR2.
53. Place in 37°C/5% CO_2_ incubator O/N.

### Delivery of CRISPR components to hiPSCs

**Timing: 3–4 h**

The following procedure describes the cell preparation and transfection procedure for gRNA, Cas9 protein, +/-ssODN (see Table 7 for options) using Neon nucleofector and Neon™ Transfection System 10 μL Kit. Alternative electroporation/nucleofection protocols can also be applied with appropriate optimization. For any additional gRNAs that are transfected separately the following procedure must be scaled accordingly.

54. Add 1 mL of Geltrex Coating Solution (see materials and equipment section) to each of 3 wells of a 6WP.

***Note:*** for each transfection prepare 2 receiving wells of a 6WP, and one well for untransfected cells; in this example 3 wells (Figure 3).

55. Incubate for 1 h at 37°C/5% CO_2_ incubator.
56. Pre-warm the E buffer and R buffer from the Neon Transfection Kit.
57. Mix gRNAs and Cas9 nuclease with R buffer according to the Table 7. Total volume is 15 μL.

***Note:*** See Table S1 for nucleofection mixes for SorCS1 KO and KI.

58. Incubate at room temperature for 10–20 min.

**Table 7:** Volumes of electroporation mixture components for different options for KO and KI; X indicates the component is not added

Condition/Component	Cas9 [μL]	gRNA 1 [μL]	gRNA 2 [μL]	ssODN [μL]	R buffer[μL]
1 gRNA KO	0.5	2	X	X	12.5
2 gRNA KO	1	2	2	X	10
1 gRNA KI	0.5	2	X	3	9.5
2 gRNA KI	1	2	2	3	7

**Optional step:** For KI experiments, immediately prior to transfection, add 3 μL of Alt-R HDR modified ssODN (12.5 μM) to the above pre-assembled RNP complex.

59. Set up the Neon electroporation system for transfection.
  a. Insert the Neon® Tube into the Neon® Pipette Station until you hear a click.
  b. Fill the Neon® Tube with 3 mL of Electrolytic Buffer (use Buffer E for 10 μL Neon® pipette).
  c. Set the following electroporation conditions:
    i. Tip size: 10 μL.
    ii. Pulse voltage: 1200 V.
    iii. Pulse width: 30 ms.
    iv. Pulse number: 1.
60. Prepare 10 mL of StemFlex/CloneR2, warm to RT.
61. Dissociate the cells:
  a. Wash the cells once with room temperature (RT) PBS (-Ca, -Mg).
  b. Add 1 mL of TryplE to each of 2 wells of 6WP.
  c. Incubate for 6–7 min at RT.
  d. Triturate the cells for 3 to 5 times with P1000.
  e. Collect the cells from each well into separate 15 mL falcon tubes (tube 1 and 2).
  f. Add 2 mL of StemFlex/CloneR2 to each tube.
  g. Centrifuge the cells at 300 g for 5 min.
  h. Aspirate the media.
62. Resuspend the cell pellet in tube 2 (step 61.e) in 1 mL of media.
63. Aspirate Geltrex Coating Solution from one well of the 6WP (step 54).
64. Add 1.5 mL of StemFlex/CloneR2 to the empty well.
65. Plate 500 μL of the cell suspension from step 62 into prepared well of a 6WP; this well with untransfected cell control is well A.
66. Tap the cell pellet in tube 1 (step 61.e) gently to dislodge it.
67. Add 20 μL of R buffer (from step 56) to the cells in tube 1.
68. Add 20 μL of cell suspension to 15 μL of HiFi Cas9 RNP +/-ssODN prepared in steps 57–58.
69. Take 10 μL Neon tip with Neon® Pipette.
70. Take up 10 μL of cell/RNP suspension (from step 68).

***Note:*** make sure there are no bubbles in Neon tip after collecting cell suspension

71. Insert the tip into the Neon tube and press the start button.
72. Transfer 10 μL cell suspension to the 1 mL of StemFlex/CloneR2.
73. Repeat step 69–72.
74. Aspirate Geltrex Coating Solution from the remaining 2 wells of a 6WP (steps 54 and **65**).
75. Add 1.5 mL of StemFlex/CloneR2 into each of the empty wells.
76. Plate 500 μL of transfected cells (step 72) into each of the two wells; these are two wells with transfected cells: wells B and C.
77. Culture the cells in incubator at 37°C/5% CO_2_/5% O_2_.

**Optional step:** For SNP KI incubate cells at 32°C/5% CO_2_/5% O_2_ for 48 h to improve HDR efficiency up to 2 fold.^24^

**Optional step:** For SNP KI use 1 μM AltR HDR enhancerV2 for 48h after transfection. For example, supplement 2 mL of Stem Flex/CloneR2 media with 2.8 μL of IDT HDR enhance v2 (1 μM) per 1 well of 6 well plate.

***Note:*** we recommend to also use HDR enhancer in combination with ‘cold-shock’. We observed ∼ more than 7-fold increase in KI-score value when using HDR enhancer (n = 1).

78. After 48 h, change media to StemFlex media without Clone R2.

***Note:*** after removal of CloneR2 cell morphology will change; while CloneR2 is present in the media cells appear slightly more spread out and have less rounded morphology (more spindle-like). However, the overall morphology of hiPSCs remains like that of typical human pluripotent stem cells, characterized by colonies with well-defined borders and individual cells with a high nucleus-to-cytoplasm ratio.

### Validation of editing efficiency in bulk sample

**Timing: 24 h**

Gene editing efficiency can be assessed in the bulk sample based on the distribution of the sequencing chromatogram from ∗.ab1 files. Freely available online deconvolution software compares sequencing obtained from unedited cells with sequencing from mixed edited cell population and discerns the percentages of different indels and/or HDR incorporation. The most commonly used tools include: Synthego ICE Tool (https://ice.synthego.com/#/) and TIDE (https://tide.nki.nl/). Such tools are not exact but offer good approximation of the editing efficiency, which in turn can help to decide on the number of clones to be screened. For example, if predicted editing efficiency is 50%, screening of 30 clones is sufficient to yield 15 clones with desired modification. On the other hand, if editing efficiency is only 5% it would be necessary to screen 300 clones to arrive at the same number of edited clones.

Here we describe analysis with Synthego tool.

79. 72 h after transfection, harvest the cells and isolate gDNA from well A (step 65) and well C (step 76). See Before you begin gDNA isolation.
80. Continue daily media changes with StemFlex in well B (step 76) until the cells reach ∼75% confluency.
81. When the cells are 75% confluent in well B, freeze the cells:
  a. Aspirate media.
  b. Wash the cells once with 0.5 M EDTA, pH 8.0.
  c. Add 1 mL of 0.5 M EDTA, pH 8.0.
  d. Incubate at 37°C for 1–3 min.
    ***Note:*** make sure that the colonies begin to detach but not completely.
  e. Aspirate EDTA slowly.
  f. Add 2 mL of BambankerTM freezing medium to the well.
  g. Using a 5 mL pipette dislodge the cells gently by pipetting.
    ***Note:*** Do not pipette up and down more than 5 times not to break up the clumps.
  h. Transfer the cell suspension into 2 cryovials.
  i. Place the cryovials immediately into the freezing container and at −80°C overnight.
  j. Transfer the cells to a liquid N2 tank the next day.

**Optional Step:** If the editing efficiency is evaluated (Steps 82–88) before the cells reach 75% confluency, the cells can be plated directly for single cell clone isolation (see section single cell clone isolation) without freezing. Step 81 can be omitted. Alternatively, cells remaining after clonal plating can be frozen.

82. Use gDNA A and C from step 79 to perform PCR using conditions chosen in step 37.

***Note:*** use 30 μL reaction volume.

**Optional step:** Resolve 10 μL of each of the PCRs (A and C) by 1%–2% agarose electrophoresis to make sure the PCR generated bands of expected size.

83. Purify remaining 20–30 μL of each reaction using PCR purification Kit.
84. Perform Sanger sequencing on the PCR products (A and C) with forward primer, the same one used for PCR.
85. To estimate bulk editing efficiency, go to: https://ice.synthego.com/
86. Follow the online prompts and input:
  a. Name of the analysis.
  b. gRNA sequence (up to 3 gRNAs) without the PAM sequence.
  c. ∗.ab1 file of the control cells (A).
  d. ∗.ab1 file of the transfected cells (C).
87. Submit the files for deconvolution analysis.
88. The analysis will provide:
  a. alignment of the control and edited sample traces (Figures S3A and S3C).
  b. success rates for KO, KO score, defined as proportion of indels that indicate a frame shift or are 21+bp in length (Figure S3B) or KI efficiency, a KI score, defined as proportion of sequences that indicate the knockin insert (Figure S3D).
  c. Indel and/or HDR distribution.
  **Optional Step:** When performing larger excisions (i.e., whole exon), it might be sufficient to assess the efficiency of editing by resolving PCR products on the gel.

### Single cell clone isolation

**Timing: 2 weeks**

Once the editing efficiency is assessed, cells can be plated for single cell clone isolation. Here we describe the single cell cloning using isoCell single cell cloning platform. However, other methods can be applied, i.e., limiting dilutions and clone picking. The following procedure is described for plating one cloning plate (256 individual wells), with expected outgrowth efficiency of ∼40%, resulting in ∼100 individual clones. The procedure is as described^4^ with modifications.

**Optional Step:** If the cells were not frozen but remain in the well B (**Step 80)**, omit Step 81 and Step 89. Proceed directly with Step 90.

89. 48 h before planned single cell seeding (Day -2), thaw cells frozen in step 81:
  a. Add 1 mL of Geltrex Coating Solution (see materials and equipment section) to 1 well of a 6WP.
  b. Incubate for 1 h at 37°C/5% CO_2_ incubator.
  c. Remove cryovial from LN2 and place on dry-ice.
  d. Prepare 10 mL of StemFlex supplemented with 650 μL of CloneR2 (1:20).
  e. Aliquot 8 mL of StemFlex/CloneR2 to a conical tube.
  f. Quickly thaw frozen cells by submerging the vial in 37°C water bath.
  g. Wait for almost all the ice to melt.
  h. Transfer the cell suspension slowly to the conical tube using P1000.
    ***Note:*** do not pipette up and down excessively
  i. Centrifuge the cells at 300 g for 3 min.
  j. Aspirate the supernatant.
  k. Resuspend the cells in 2 mL of StemFlex/CloneR2.
  l. Aspirate Geltrex Coating Solution from the well.
  m. Gently plate 2 mL of cell suspension in the well.
  n. Place in 37°C/5% CO_2_ incubator O/N.
  o. After 24 h, perform media change with StemFlex/CloneR2.
90. Prepare the GRID on a IsoCell dish for single cell plating (Day 0).
  a. Add 2 mL of StemFlex media to provided 60 mm cell culture dish.
  b. Incubate at 37°C for 5–10 mnts.
  c. Aspirate StemFlex.
  d. Carefully add 2 mL of FC40STAR over the inner rim of the dish.
  e. Align the dish on the isoCell.
  f. To fabricate a GRID, select the ‘GRID’ program (‘Isolate’ menu tab).
  g. Follow prompts on the isoCell display.
91. Perform single cell plating:
  a. Perform single cell passage on the transfected cells from Step 80 or Step 89:
    i. Perform single cell passaging following the Steps 49–50.
    ii. In a 1.5 mL Eppendorf tube, prepare 500 μL of cell suspension in StemFlex/CloneR2 at a concentration of 7,500–10,000 cells/mL.
    iii. Add 4.4 μL of iMatrix-511 (0.025 μg/cm^2^).
    iv. Place the tube with cells on the isoCell.
  b. To plate single cells into GRID chambers run the ‘Plate’ program (‘Isolate’ menu tab).
  c. Follow prompts on the isoCell display.
92. After the cells are dispensed into the GRID, incubate the plate at 37°C for 20–30 min.
93. Using isoHub, identify and select the chambers with single cells.

**Figure 4:**
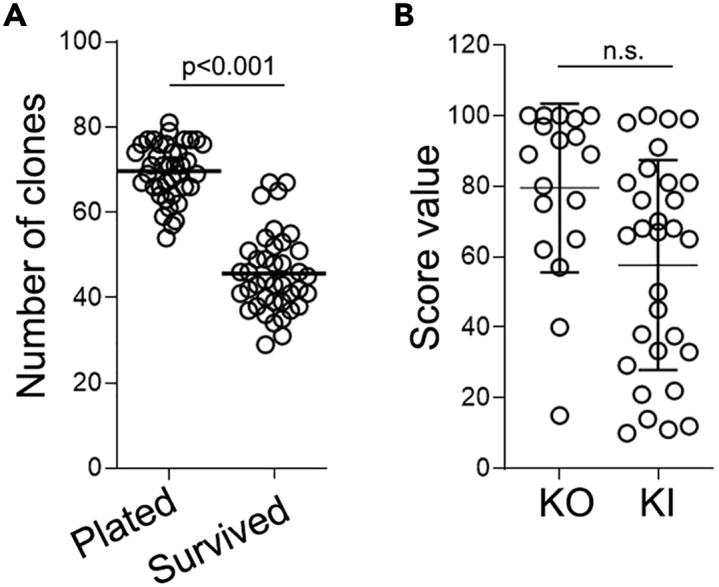
ASSURED protocol results in high editing and cloning efficiency (A) Scatter dot plot showing the total number of clones per grid plate plated using IsoCell robot. Bar: mean. (B) Scatter dot plot showing the KO or KI scores for individual editing’s. Each dot represents one editing project (one gene, one cell line). Bar: mean, error bars: SD. N.s. = not significant.

***Note:*** on average 70 chambers are expected to contain a single cell (Figure 4A).

94. Incubate cells at 37°C, 5% CO_2_, 5% O_2_ overnight.

**Optional Step:** If isoHub is not available, perform the identification of chambers with single cell manually and input the data into isoCell using the ‘Input’ program (‘Isolate’ menu tab).

95. 72 h after plating (Day 3), fill GRID chambers containing a single cell with StemFlex/CloneR2 medium using the ‘Fill’ program (‘Culture’ menu tab).
96. Follow prompts on the isoCell display.
97. Incubate cells at 37°C, 5% CO_2_, 5% O_2_ for 48 h.
98. Perform daily media change starting from Day 5 StemFlex following the ‘Feed’ program (‘Culture’ menu tab).
99. If colonies cover around ¼ of a GRID chamber (typically on Day 7 or 8) the clones should be transferred to 96-well plates.

**CRITICAL:** do not let the chambers reach more than ½ of the surface area, if cells overgrow the media volume is not sufficient to support cell survival

100. Prepare two 96-well plates for each 96 clones to be harvested.
  ***Note:*** Each clone will be split into 2 wells of a 96WP to create mirror plates. Therefore, for each clone ready to be harvested coat one well in each 96WP.
  a. Add 50 μL of Coating Solution (see materials and equipment section) to required number of 96 wells. For example, for 36 clones, coat 72 wells: 36 wells in each of the 96WPs.
  b. Incubate at 37°C, 5% CO_2_ for 1 h.
  c. Prepare StemFlex/CloneR2.
    ***Note:*** Required volume will be dependent on the number of clones, but generally each clone requires 320 μL of media. For example, for transfer of 32 clones, 12 mL of media is required.
  d. Aspirate Geltrex Coating Solution.
  e. Add 100 μL of StemFlex/CloneR2 medium to each well.
101. Detach single cell clones from the selected chambers:
  a. Select up to 16 GRID chambers containing clonal colonies for detachment.
  b. Following the instructions in the ‘Detach’ program (‘Harvest’ menu tab).
    ***Note:*** The program washes cells twice with PBS before adding TrypLE to selected GRID chambers
  c. Incubate the 60 mm dish at 37°C for 10–15 min.
    ***Note:*** Observe the cells under the microscope every 5 min to assess the detachment
  d. During this incubation, add 120 μL of StemFlex/CloneR2 medium to each PCR strip tube and place them onto the isoCell PCR strip holder.
102. Extract single cell clones from the detached wells:
  a. Use an isoHub or bright field microscope to ensure that cells in GRID chambers detached (rounded up cell morphology).
  b. Perform the ‘Extract’ program (‘Harvest’ menu tab) on selected GRID chambers.
    ***Note:*** For each chamber the isoCell aspirates the detached cell and plate each clone into one PCR tube.
  c. Distribute 60 μL of cell suspension from each clone into 2 wells of each of the 96WP (prepared in Step 100)
  d. Incubate cells at 37°C, 5% CO_2_, 5% O_2_ for 48 h.
103. Perform media change using StemFlex/CloneR2 (150 μL/well) every other day for 96 h.

***Note:*** One of the 96WP should be ready for DNA extraction at 48–72 h, for DNA extraction with one plate continue to **Step 105**.

104. With the second 96WP, after 96 h continue with daily media changes using StemFlex without CloneR2.

**CRITICAL:** If **Steps 104–108** are completed before the clones are 70% confluent proceed to **Step 117**, otherwise continue to **Steps 109–119** to avoid overgrowing of the clones.

### Screening for positive edited clones

**Timing: 24–48 h**

PCR amplification and Sanger sequencing can be performed using diluted crude whole cell lysates. We recommend using Phire Animal Tissue Direct PCR. Cells are usually ready for gDNA extraction∼48 h after plating (Step 102) or when confluency is 25%–50%.

105. Prepare appropriate volume of the PhireDNA lysis buffer according to manufacturer instructions; 25 μL per well; for example, for 40 clones prepare 1 mL of lysis buffer.

***Note:*** Cells in only one of the 96WP will be lysed, the clones from the other plate will be frozen see **Steps 110–117**.

106. Perform DNA extraction:
  a. Aspirate the media from 96WP.
  b. For each 96-well plate to be lysed, add 25 μL of DNA lysis buffer from Phire Animal Tissue Direct PCR Kit.
  c. Incubate for 2 min at RT.
  d. Transfer samples to a 96 well PCR plate.
  e. Incubate for 2 min at 95°C in thermocycler.
  f. Cool down to RT.

***Note:*** Use 1 μL for PCR amplification. If the sample is viscous, dilute the samples 2-fold in RNAse/DNAse free water.

107. Perform PCR on the gDNA extracted from clones as optimized in step 37.
108. Perform Sanger sequencing on PCR products using forward primer.
109. Perform KO or KI analysis as described step 85–88 (Figures S4–S5).

**CRITICAL:** Carefully analyse sequencing and Synthego ICE results for analysis of the clones. Some clones will remain WT (Figures S4A and S5A), some clones will be heterozygous with different repair for each allele (Figures S4B and S5B), and some clones will be homozygous for the intended modification (Figures S4C and S5B). For more details see expected outcomes section.

### Freezing of clones in 96 WP

**Timing: 1 h**

Clone freezing in 96WP allows for a brief pause for the PCR screening to be completed. It is not recommended to store clones in this format for extended period of time, as 96WP cannot be transferred to LN2.

110. Aspirate medium using a multichannel aspirator.
111. Add 20 μL of 0.5 mM EDTA, pH 8.0 to each well.
112. Incubate for 1–2 min at RT.

***Note:*** do not wait until the colonies are completely detached.

113. Quickly aspirate EDTA with a multichannel aspirator.
114. Dispense 100 μL of Bambanker freezing medium per well.
115. Close the lid of the plate and secure it with tape.
116. Wrap the plate in a couple of layers of paper towels or other insulation.

***Note:*** plate can also be placed in a Styrofoam box.

117. Place the wrapped plate immediately in the −80°C freezer.

***Note:*** as transfer to liquid N2 is not possible, the cells in the plate can only be stored short term (up to 6 weeks).

### Preparation of seed banks

**Timing: 1 week**

As genetic manipulation, clonal selection and expansion generates selection pressure and can accelerate emergence of unwanted genetic instability, we strongly recommend performing a small screen to select only genetically stable clones prior to further pluripotency characterization. The following protocol describes thawing, expansion, and Generation of seedbanks from 5–6 positive clones. This procedure allows for faster turn over in case some of the clones have undesirable karyotype profiles.

118. Coat the receiving 6WP plate with Geltrex Coating Solution (see materials and equipment section).
119. Incubate at 37°C for 1 h.
120. Prepare StemFlex/CloneR2.

***Note:*** Volume of media needed is dependent on the number of 96WP wells being thawed. For example, for 6 clones prepare 12 mL of media.

121. Aspirate Geltrex Coating Solution from the receiving plate.
122. Add 1.5 mL of StemFlex /CloneR2 to the wells of the receiving plate.
  **Optional step:** If the cells were not frozen after Step 104, passage each well.
  a. Aspirate media from the well.
  b. Wash the cells once with 100 μL of 0.5 M EDTA, pH 8.0.
  c. Add 100 μL of 0.5 M EDTA, pH 8.0 to the well.
  d. Incubate at 37°C for 3–5 min.
  e. Aspirate EDTA slowly.
  f. Proceed with Step 125.
123. Remove 96WP from −80°C freezer and place it in 37°C bead bath or on the prewarmed pad.
124. Once Bambanker media in the 96WP starts to thaw transfer the plate to the biosafety cabinet.
125. Add ∼120 μL of prewarmed StemFlex/CloneR2 to the desired well.
126. Gently mix by pipetting.

***Note:*** each well should contain about 100 μL of Bambanker freezing media (see **Step 113**)

127. Transfer the entire volume from one well of the 96WP to one well of the receiving 6WP plate.
128. Repeat steps 126–128 for all desired wells/clones.
129. Incubate cells at 37°C, 5% CO_2_.
130. After 24 h perform ½ media change with StemFlex/CloneR2.
131. 48 h after thawing, perform full media change with StemFlex media.
132. Continue media changes until the cells reach 70% confluency.
133. Coat one full 6WP per clone with Geltrex Coating Solution (see materials and equipment section).
134. Perform 1:6 cell passage using EDTA.
135. Continue daily media changes until the cells reach 70% confluency.
136. For each clone, freeze the cells as described in step 81 (1 well per cryovial).

**CRITICAL:** Following gene editing pipeline the resulting clones should be subjected to standard iPSC quality control to ensure that no genetic abnormalities occurred during the editing and cloning process. We recommend expanding the selected clone, freezing a bank (∼50 vials) and performing SNP array analysis and karyotyping to assess genetic stability. Whenever possible, we recommend banking at least two edited clones. Additionally, a routine assessment of morphology and expression of markers of undifferentiated cells should be conducted. Confirmation of genetic modification by PCR and Sanger sequencing or using NGS should also be performed on final bank. We also highly recommend assessing the integrity of the 5–10 top off-target sites for each of the gRNAs used.

## Expected outcomes

Design and selection guidelines for gRNA and HDR template described above are universal and should allow for successful targeting of most of the genomic loci. Primer optimization (Steps 18–22, 33–42) prior to editing experiment allows us to reduce time and effort during clone screening and ensures good quality of sequencing for clone validation. Introduction of freezing pause steps (Step 81) allows for more flexibility.

We have successfully applied the ASSURED protocol to target 32 gene loci across 22 different iPSC lines (Figure 4B). On average, we obtained KO-score of 79.5 ± 23. KI efficiency is generally lower than for KO, with an average KI-score of 57.5 ± 27 (Figure 4B). The optimized cloning method using IsoCell resulted in ∼70 single cell containing chambers per one 256-chamber plate as scored on the day of plating (Step 92, Figure 4A) and out of those on average 46 (65%) clones survive (Figure 4A). The lowest KO and KI scores we encounter were 15 and 10 respectively (Figure 4B). Despite that, due to relatively high cloning efficiency, up to 5 clones were obtained from just one 256-chamber plate.

## Limitations

Although we have not yet encountered such a case, for certain genetic loci in combination with specific hiPSC lines it might be impossible to identify a suitable gRNA or perform successful HDR. In such cases, excisions of larger fragments (i.e., whole exon) and subsequent repair with larger templates might be an option. Some cell lines might be more sensitive to single cell cloning, requiring optimization of cloning strategy. In cell lines with higher susceptibility to spontaneous differentiation, it might be necessary to generate seed banks from more clones, to identify the most stable clones.

Finally, targeting genes controlling proliferation or cell survival, especially for KO, might result in growth disadvantage or cell death of the successfully edited clones. Therefore, targeting certain genes might not be biologically feasible.

## Troubleshooting

### No gRNAs directly at the editing site (steps 2–17)

No gRNA in the immediate proximity to the target site, or existing gRNAs have high off-target or low on target scores.

### Potential solution

Identify gRNAs targeting upstream and downstream of the target site. Design longer HDR and perform large KI. It is advisable to perform gRNA cutting efficiency testing prior to including HDR in the transfection.

### Poor cell survival after nucleofection (steps 61–80)

Cell lines do not attach after nucleofection or die in subsequent days.

### Potential solution

Some iPSC lines are sensitive to culture condition changes or exposure to certain chemicals (AltR HDR enhancer, etc.) and therefore pre-adapt the cell line to the Stem flex media conditions and optimize HDR enhancer concentration. Additionally, optimization of cell numbers used or the voltage for nucleofecting might be necessary.

### Low gRNA cutting efficiency (steps 85–88)

After bulk analysis there are few indels and/or low KO score.

### Potential solution

On-target scores for gRNAs provided by CRISPR design software are derived purely in silico. Therefore, it is possible that *in vitro* the gRNAs cutting efficiency is not as high as predicted. We recommend selecting additional gRNAs in the region, if available, or adapting the strategy as described for ‘No gRNAs directly at the editing site’.

### High gRNA cutting efficiency but low HDR efficiency (steps 85–88)

Low HDR efficiency.

### Potential solution

If not done previously, add HDR enhancers. Additionally, redesign the HDR template by changing the asymmetry ratio, target strand or length of homology arms. Use HDR template mixes; mix HDR templates with different symmetries, or homologous to either of the strands.

### Low cloning efficiency (steps 91–99)

Very few clones survive the single cell cloning step.

### Potential solution

Optimize the culture conditions for the specific iPSC line. Some iPSC lines are more sensitive to single cell growth conditions. It might be necessary to test other media and/or coating of the grid chambers.

### No clones positive for the desired modification (steps 107–109)

Editing efficiency was high based on bulk sequencing, but no positive clones were identified after clonal seeding.

### Potential solution

Designed edit results in growth disadvantage or cell death. Reevaluate biological function of the target gene and check if there is no indication that gene modification can be lethal or detrimental to cell proliferation. In specific case of iPSCs alterations to pluripotency genes may lead to undesirable differentiation.

### Too many KI homozygous clones (steps 107–109)

The project requires heterozygous and homozygous clones, but all clones identified with the HDR incorporation are homozygous.

### Potential solution

Repeat the experiment including WT HDR template. Use 1:1 mixture of WT and mutant templates to increase the chance of obtaining heterozygous clones.

### Unexpected phenotype (steps 129–135)

Decreased proliferation, excessive cell death or differentiation.

### Potential solution

See ‘No clones positive for the desired modification.

## Resource availability

### Lead contact

Further information and requests for resources and reagents should be directed to and will be fulfilled by the lead contact, Sebastian Diecke (Sebastian.Diecke@mdc-berlin.de) and technical contacts, Katarzyna Anna Ludwik (Katarzyna.ludwik@bih-charite.de) and Narashima Telugu (NarasimhaSwamy.Telugu@mdc-berlin.de).

### Materials availability

No unique reagent was generated in this study.

## Data Availability

No unique datasets or data were generated in this study.
