# Supplementary material for: ASSURED-optimized CRISPR protocol for knockout/SNP knockin in hiPSCs
Source: STAR Protoc. 2023 Jul 22;4(3):102406. doi: 10.1016/j.xpro.2023.102406 (PMC10382939; doi:10.1016/j.xpro.2023.102406)
Supplement: Document S1. Figures S1–S5 and Tables S1 and S2 [file mmc1.pdf]

Figure S1. How to choose primers. Related to Steps 20 and 37. A) Alignment of 2 primer pairs for amplification of exon 4 region in SorCS1 gene. Created with SnapGene B) Alignment of 2 primer pairs for amplification of exon 25 region in SorCS1 gene. Created with SnapGene C) Example gel electrophoresis of PCR products obtained from parental iPSC gDNA using primer pairs from (A) with various polymerases and annealing temperatures. Lanes 1 and 14: size marker; 5, 9, 13 ,18 ,22 ,26: empty; 2, 6, 10, 15, 19, 23: annealing temp. 60ºC; 3, 7, 11, 16, 20, 24: annealing temp. 62ºC; 4, 8, 12, 17, 21, 25: annealing temp. 64ºC. D) Example gel electrophoresis of PCR products obtained from parental iPSC gDNA using primer pairs from (B) with various polymerases and annealing temperatures. Lanes 1 and 14: size marker; 5, 9, 13 ,18 ,22 ,26: empty; 2, 6, 10, 15, 19, 23: annealing temp. 60ºC; 3, 7, 11, 16, 20, 24: annealing temp. 62ºC; 4, 8, 12, 17, 21, 25: annealing temp. 64ºC.

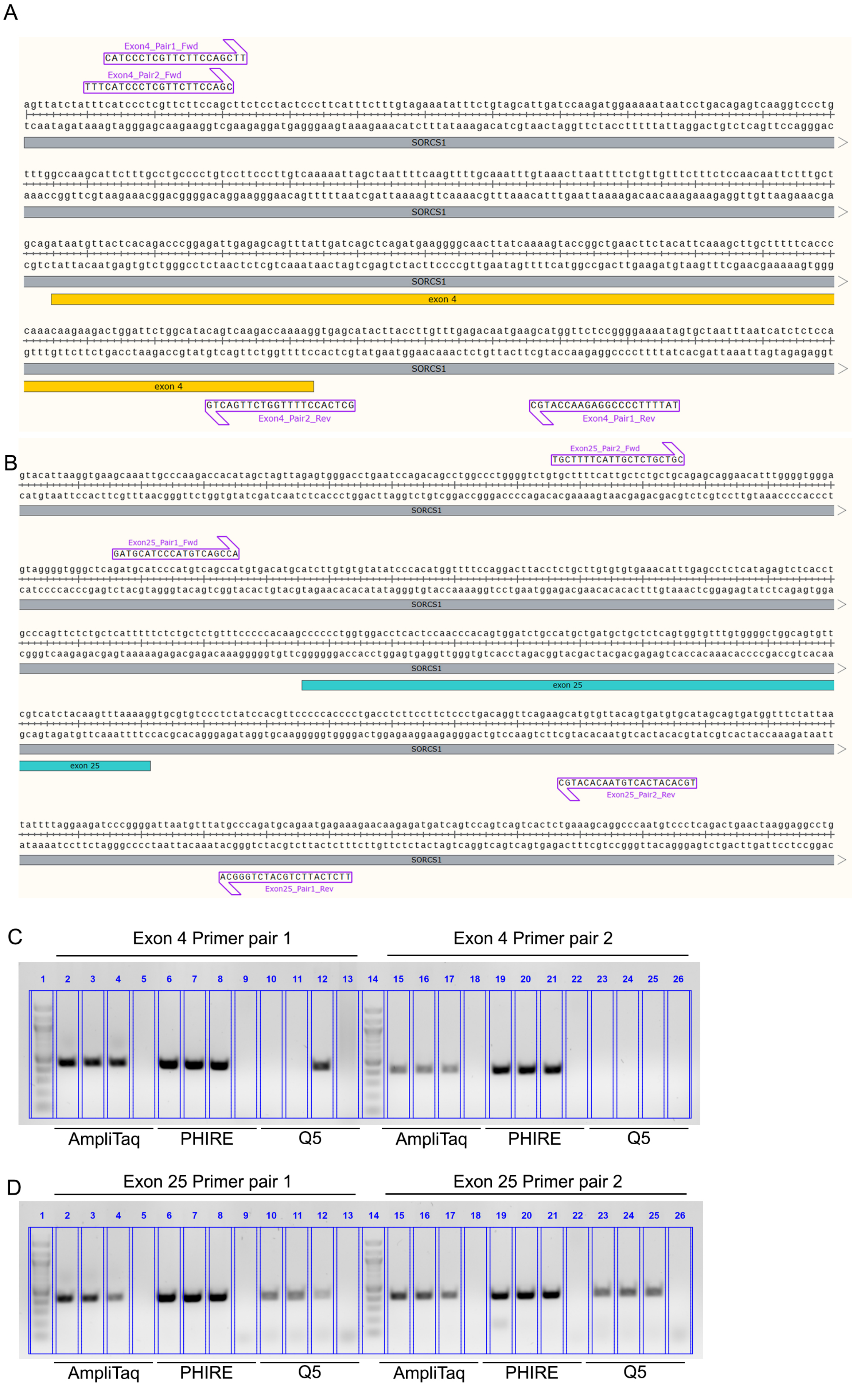

Figure S2. SN variation between different iPSC lines. Related to Step 41. A) Example chromatograms of POMC gene fragment sequencing from two iPSC cell lines. Blue box indicates gRNA sequence, pink highlights PAM sequence, yellow marks SN variation inside gRNA seed sequence. Created with SnapGene

A

BIHi005-A

BIHi261-A

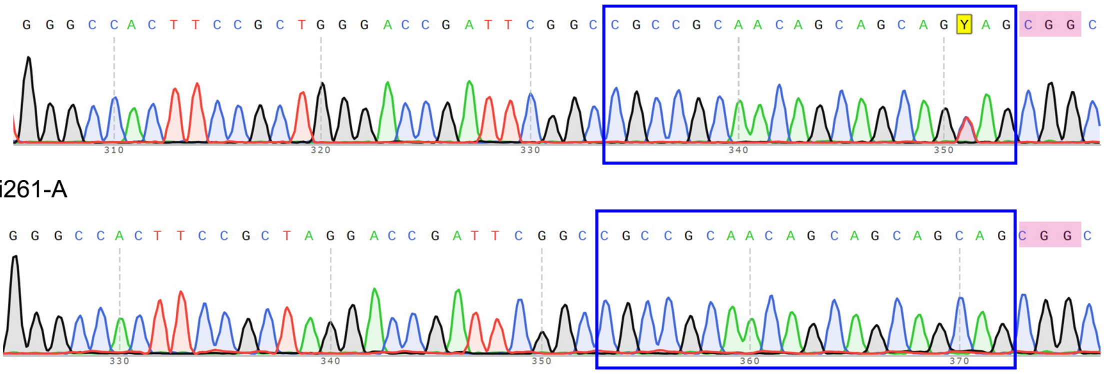

Figure S3. Gene editing efficiency assessment in bulk sample. Related to Step 88. A) Example trace analysis obtained from control or edited bulk sample sequencing from SorCS1 exon 4 KO targeting (analyzed by Synthego ICE Tool). B) Indel distribution analysis obtained from deconvolution of traces from (A). Red box indicates the KO-score. C) Example trace analysis obtained from control or edited bulk sample sequencing from SorCS1 exon 25 KI targeting (analyzed by Synthego ICE Tool). D) Indel distribution and HDR incorporation analysis obtained from deconvolution of traces from (C). Red box indicates the KI score.

A SorCS1 Exon 4 knock-out

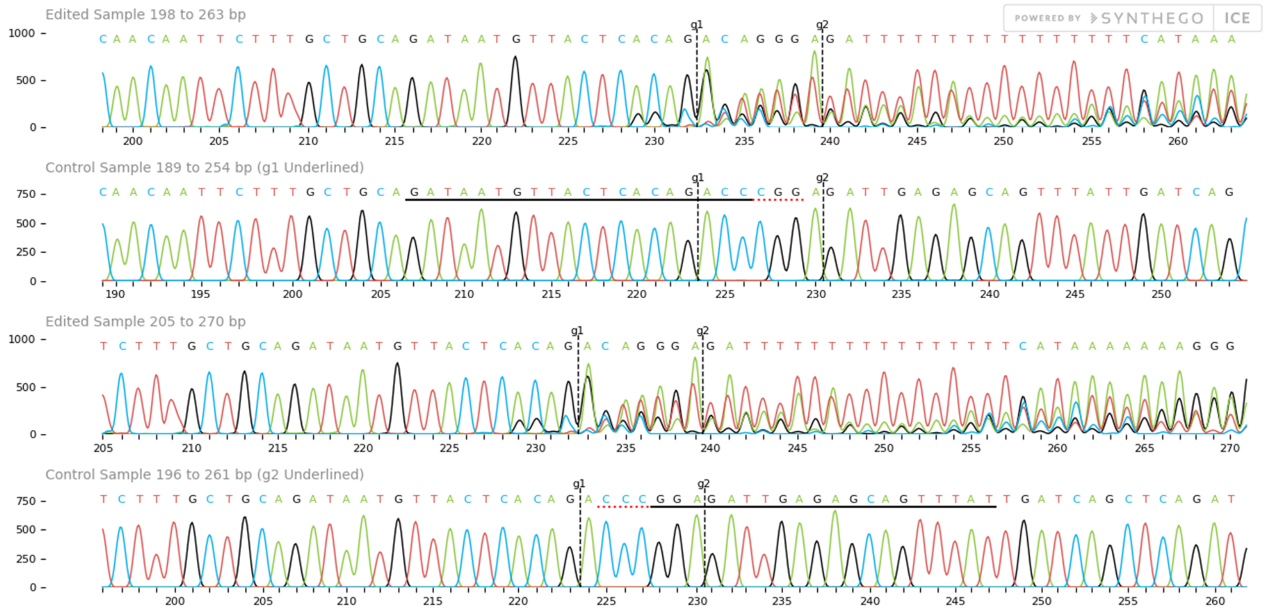

B

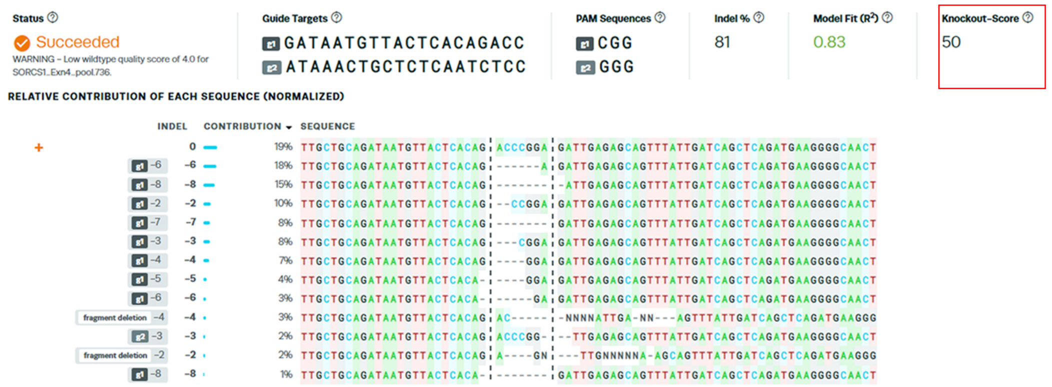

C SorCS1 Exon 25 knock-in

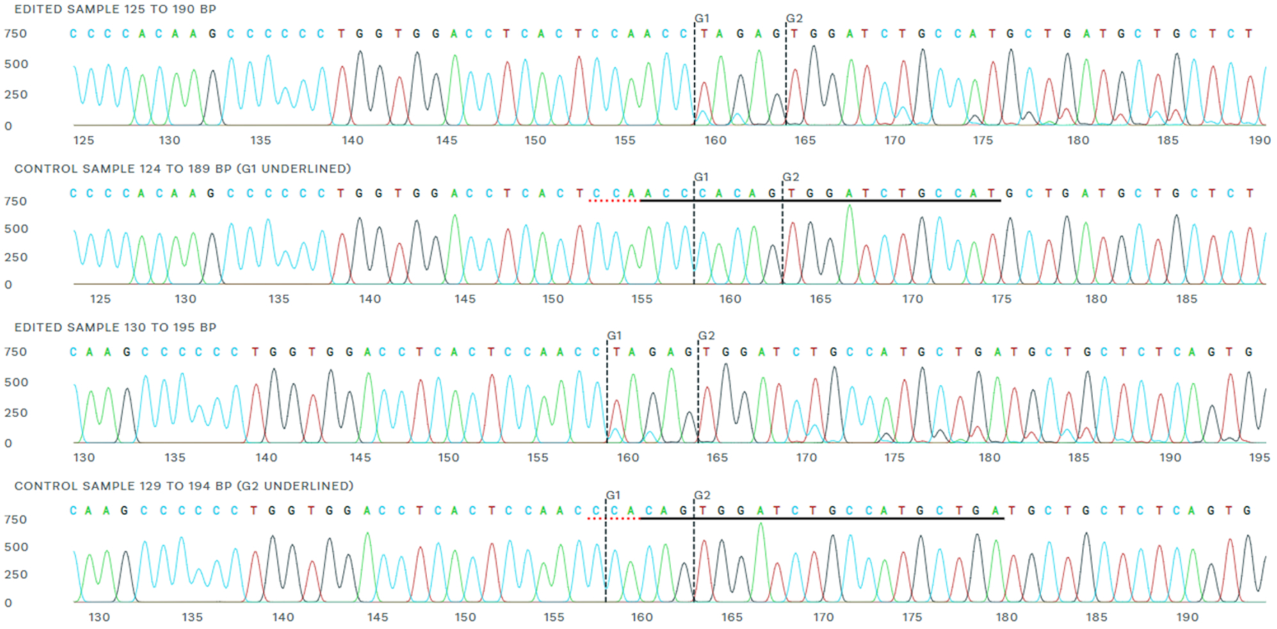

D

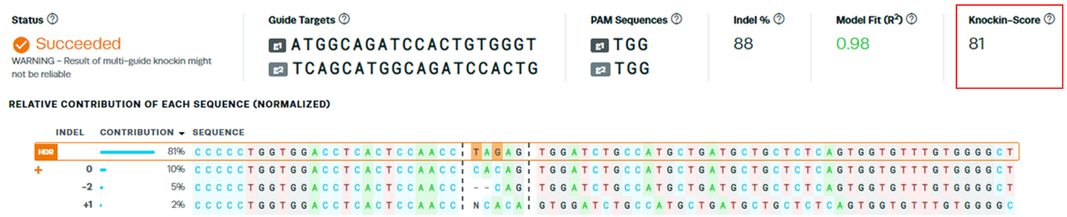

Figure S4. Analysis of individual edited clones from SorcCS1 exon 4 editing. Related to Step 109.
Trace analysis obtained from control and edited clone sequencing from SorcCS1 exon 4 KO targeting (A, B, C) (analyzed by Synthego ICE Tool).

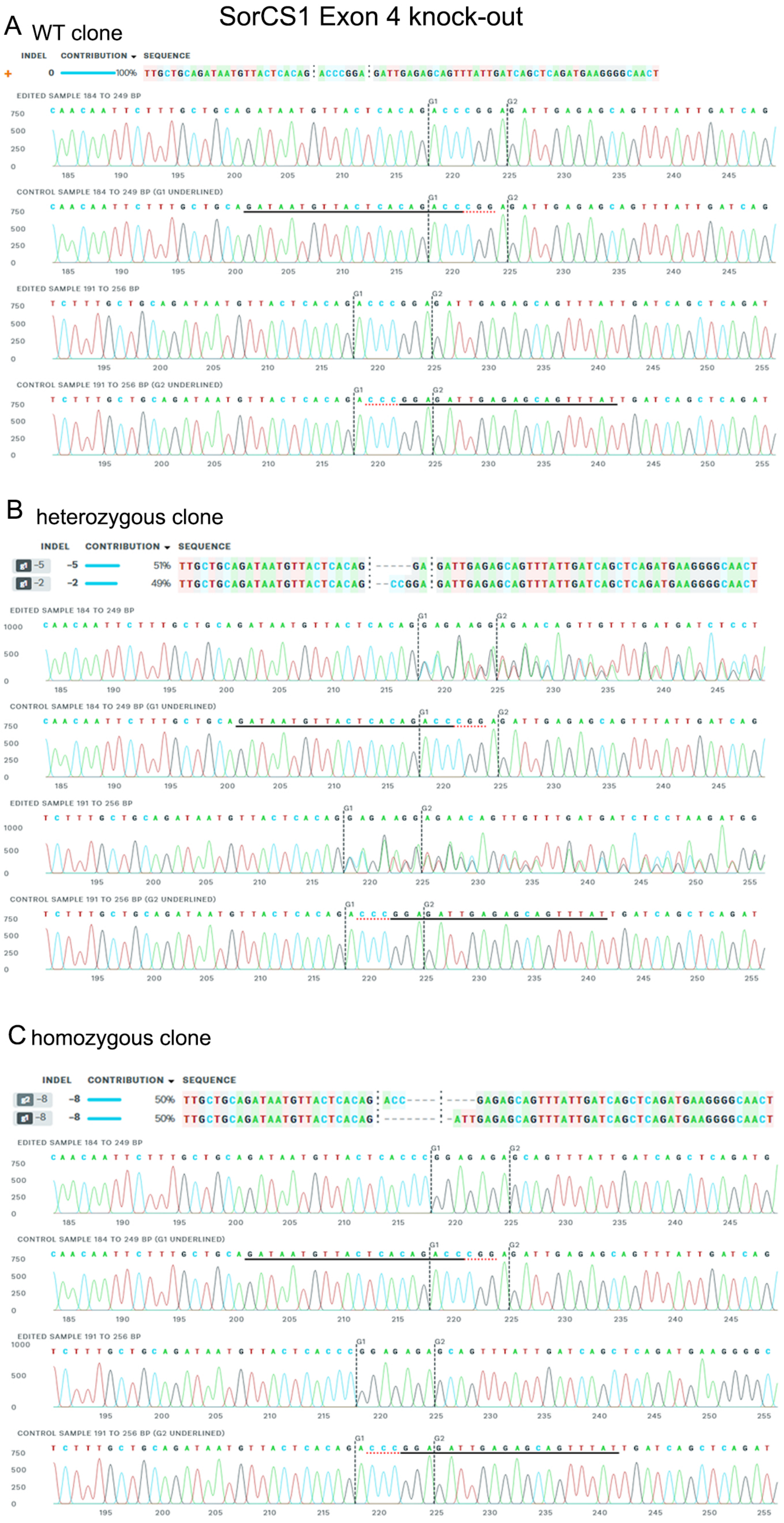

Figure S5. Analysis of individual edited clones from SorcCS1 exon 25 editing. Related to Step 109.
SorCS1 exon 25 KI targeting (A, B) (analyzed by Synthego ICE Tool).

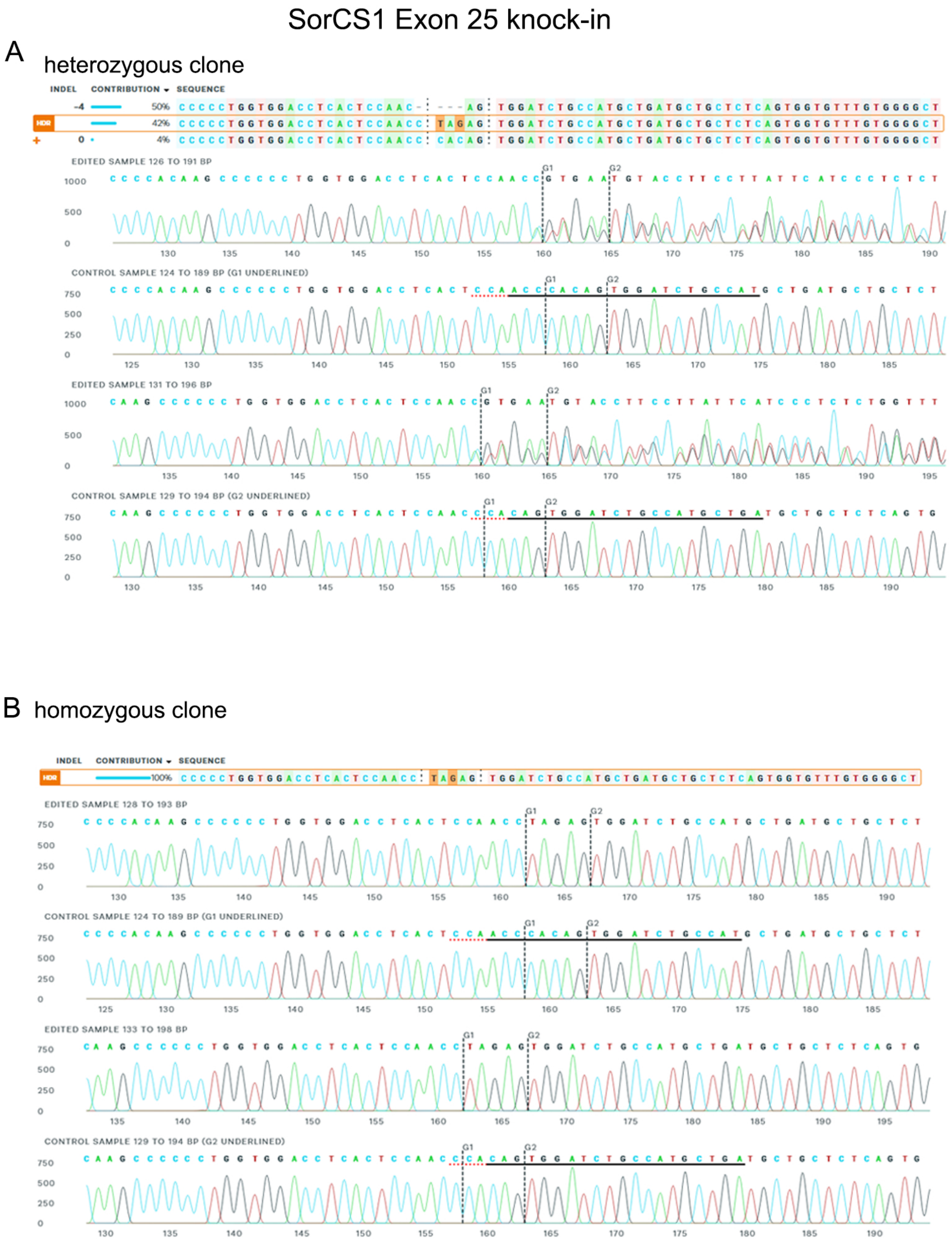

Supplementary Table S1. Volumes of RNP mixture components for Neon Transfection for SorCS1 gene KO (Exon 4) and KI (Exon 25).

Cas9 [ul]	SorCS1-Exn4-gRNA1 [ul]	SorCS1-Exn4 gRNA2 [ul]	ssODN [ul]	R buffer[ul]
1	2	2	X	8.5
Cas9 [ul]	SorCS1-Exn25-gRNA1 [ul]	SorCS1-Exn25-gRNA2 [ul]	ssODN [ul]	R buffer[ul]
1	2	2	3	5

Supplementary Table S2. Oligonucleotides, continued from Key Resource Table.

REAGENT or RESOURCE	SOURCE	IDENTIFIER
Oligonucleotides		
Alt-R® CRISPR-Cas9 gRNA, 2 nmol<br>SorCS1-Exn25-gRNA1<br>ATGGCAGATCCACTGTGGGTC	Integrated DNA technologies (IDT)	IDT predesigned and custom gRNA
Alt-R® CRISPR-Cas9 gRNA, 2 nmol<br>SorCS1-Exn25-gRNA2<br>TCAGCATGGCAGATCCACTG	Integrated DNA technologies (IDT)	IDT predesigned and custom gRNA
Alt-R™ HDR Donor Oligo, 2 nmol<br>SorCS1-Exn25-ssODN<br>GAGCCTCTCATAGAGTCTCACCTGCCCAG<br>TTCTCTGCTCATTTTTCTCTGCTCTGTTTCC<br>CCCACAAGCCCCCCTGGTGGACCTCACTC<br>CAACCTAGAGTGGATCTGCCATGCTGATG<br>CTGCTCTCA	Integrated DNA technologies (IDT)	Custom oligo
